# A Snapshot of Community Engagement in Research in the Context of an Evolving Public Health Paradigm: Review

**DOI:** 10.2196/jopm.8939

**Published:** 2018-01-17

**Authors:** Catherine Maree Holliday

**Affiliations:** ^1^ Centre for Community-Driven Research Ultimo Australia

**Keywords:** Community engagement, weight loss, paradigm shift, public health policy

## Abstract

**Background:**

Community engagement is a work in process. Researchers continue to refine the process of collaboration and look to best practice and lessons learned for guidance in this relatively new model.

**Objective:**

The aim of this study was to provide a snapshot of whether community engagement has been included in the design and implementation of research initiatives in Australia, Canada and the United Kingdom. The secondary aim is to identify the underlying themes present, to identify theories and tools that drive research.

**Methods:**

A literature search was performed to identify studies that have been conducted to reduce the weight of the general population.

**Results:**

The results of the study, which focused on the field of weight loss, indicate that scientific and technological advancements are the primary drivers of research. However, these new research initiatives have largely been undertaken in the absence of community engagement.

**Conclusions:**

The study concludes that initiatives need to adapt to a wider range of stakeholders, develop equitable community engagement platforms and take into consideration.

## Introduction

Early phases of public health focused on creating physical infrastructure, improving sanitary conditions, and fighting and containing known infectious diseases [1]. This model addressed the immediate needs of the population and set the fundamental basis for modern public health systems. Further movements, particularly towards the end of the 20^th^ century, addressed the role of individual behavior on noncommunicable diseases and premature death and focused on disease prevention [2]. A modern public health emerged in the 1990s with a consensus in the international community that health promotion guided by the Ottawa Charter principles constituted public health [1]. The significance of the new public health was that it recognized health as a key determinant of the quality of life of individuals and specific populations. It incorporated elements from previous models to create a new movement with a more unified, community-based and interconnected path between the many components of public health [3,4].

Modern public health continues to evolve and is responsive to globalization, and political and physical environments. As with early phases of public health, modern public health still places importance on physical infrastructure; however, the aim is to enhance its value and effectiveness with the addition of social support and acknowledgement of behavioral factors; creating a more holistic, intersectorial approach to health issues [5]. The beginning of this century has seen a further extension of modern public health where factors that are not traditionally health related, such as environmental sustainability and intellectual property, are also taken into consideration when reacting to health issues and developing initiatives [6].

While there have been shifts towards more social movements to improve the health of communities, they are still primarily expert driven, top-down initiatives [4]. Community engagement is a work in process. Health professionals and researchers continue to review and refine this process of collaboration and look to best practice and lessons learned for guidance in this relatively new model [7,8]. The aim of this study was to provide a snapshot of whether community engagement has been included in the design and implementation of research initiatives in Australia, Canada and the United Kingdom. The secondary aim is to identify the underlying themes present, to identify theories and tools that drive research.

For the purpose of this study, the field of nutrition, specifically initiatives that aim to support weight loss in a general population, will be investigated. The field of weight loss was selected as there is a growing, worldwide effort to address the impact of the increasing incidence, mortality and cost to society of overweight and obese populations. It was also selected as eating is an everyday activity and it can be reasonably expected that communities are involved in research within this field. Furthermore, while the outcomes of weight loss interventions have been reviewed, [9-16] there is little evidence on how communities have been engaged in research and the trends driving new research initiatives.

## Methods

The lead researcher performed a literature search to identify studies that have been conducted to reduce the weight of the general population. The study covered a number of key areas: public health, nutrition, health promotion, and sociology. For this reason, the lead researcher used the PubMed database to collect sources from each sector needed to achieve a balanced and comprehensive result.

The broad search included the title/abstract search terms “weight loss” and “intervention”, excluding the Medical Subject Headings “Surgical Procedures,” “Operative,” and “Drug Therapy,” with the inclusion criteria set to randomized controlled trials, studies published between 2000 and 2014, and language in English. The search restricted studies to those conducted in Australia, Canada and the United Kingdom as these comprise countries with similar public health systems.

Studies excluded were those not implemented in Australia, Canada or the United Kingdom; if the study focused on a subpopulation with a specific disease or condition; studies that included a pharmacological intervention; and studies that did not have an outcome or measurement of weight loss.

The lead researcher classified the included publications according to the focus of the intervention and grouped these into themes. This was achieved by determining the theory or element that the interventions tested within each study. The lead researcher conducted the literature search and process of classification twice to assure accuracy and consistency of classification.

The lead researcher recorded the number of studies in each theme and used this information to rank themes in an effort to understand the drivers or factors that influence the development of research initiatives. The lead researcher ranked the theme that yielded the most studies first, and the theme that yielded the least studies last.

To understand the level of community engagement included in the studies, the lead researcher reviewed each study and recorded indicators of community engagement in relation to (1) study development (whether the research group engaged a consumer or patient group/representative in the development of the protocol before the intervention was finalized), (2) future implementation (whether consumer or patient engagement is recommended as part of next steps or future work) and (3) acknowledgement (whether the research group acknowledged the contribution of participants in the study). It is important to note that acknowledgement on its own may not function as an indicator of community engagement; however, it has been included as a gauge that may be used in future studies to measure whether there is an increase in basic acknowledgment of participants in studies.

## Results

**Figure 1:**
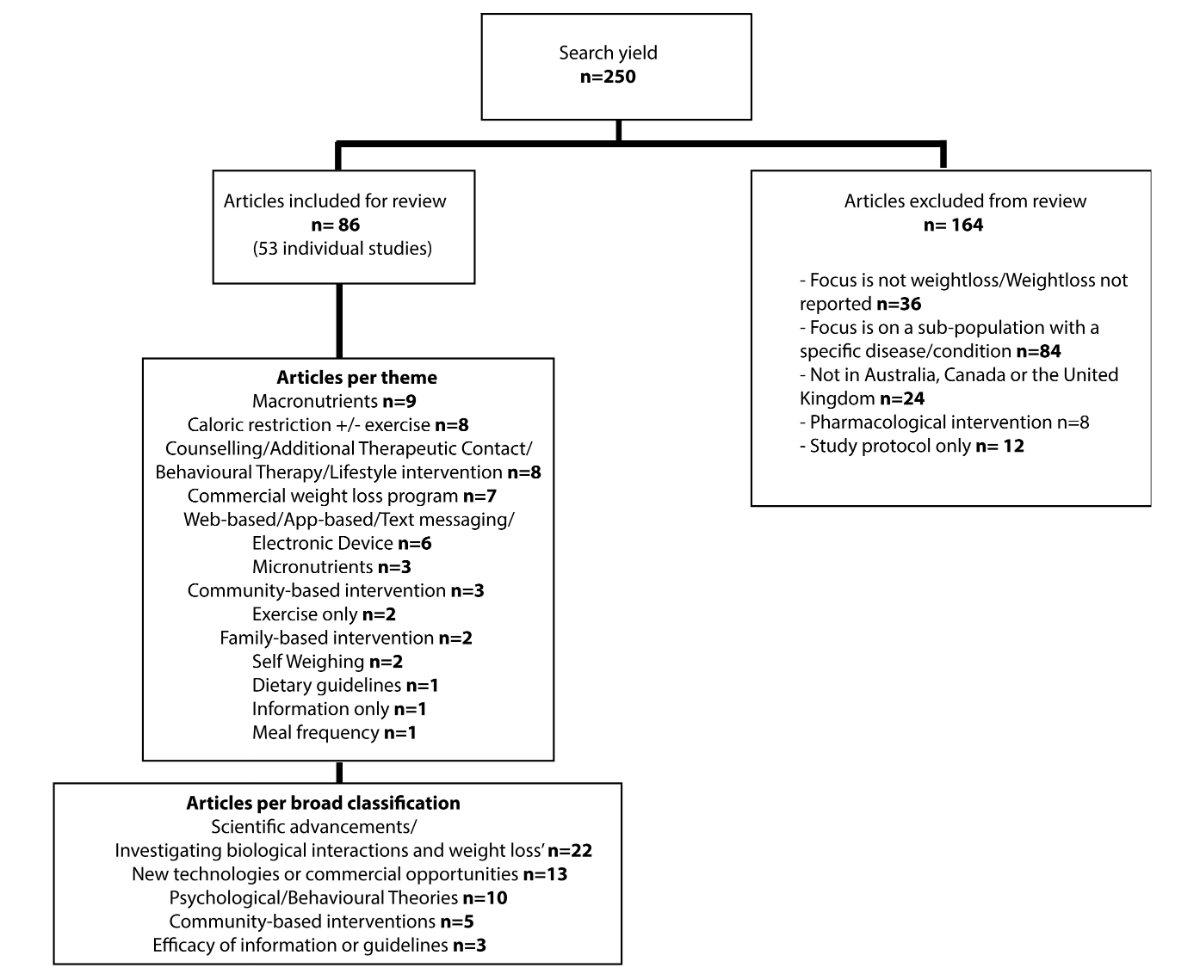
Search yield for study literature search.

The initial search of studies between 2000 and 2014 yielded 250 publications. 164 articles did not meet the inclusion criteria and 86 publications representing 53 individual studies met the inclusion criteria. See Figure 1.

Following a review of all studies, the lead researcher identified 13 classification groups and then ranked studies within each classification (see Table 1). This resulted in nine studies (17.0%) investigating “Macronutrients” and weight loss, which was the most common theme, followed by interventions that tested “Caloric restriction +/- exercise” and “Counseling/Additional Therapeutic Contact/Behavioral Therapy/Lifestyle intervention” (n=8,15.5% respectively), “Commercial weight loss programs” (n=7, 13.2%) and “Web-based/App-based/Text messaging/Electronic Device” (n=6, 11.3%). The remainder of the themes, including “Community-based interventions”, had three or fewer studies. The number of studies listed within each of the categories ranged from nine to one (see Table 2).

The lead researcher then identified five broad classifications resulting in 22 studies (41.5%) responding to “Scientific advancements/Investigating biological interactions and weight loss;” 13 studies (24.5%) responding to “New technologies or commercial opportunities;” 10 studies (18.9%) responding to “Psychological/Behavioral Theories;” five studies (9.4%) responding to theories in “Community-based interventions”; and three studies (5.7%) responding to the need to test the “Efficacy of information or guidelines.”

In relation to documented community engagement within publications, two studies (3.7%) documented evidence of community engagement in the development of the protocol, four (7.5%) noted that they would incorporate community engagement activities in future, related initiatives, and 17 studies (32.1%) acknowledged and thanked the people that participated in the study. 35 studies (66.0%) had no documented form of community engagement across the three indicators (see Table 3).

**Table 1:** Theme descriptions.

Themes	Definitions
Macronutrients	Studies that compare or investigate the interaction of macronutrients and weight loss
Caloric restriction +/- exercise	Studies that investigate caloric restriction in weight loss, some with and some without exercise
Micronutrients	Studies that compare or investigate the interaction of micronutrients and weight loss
Exercise only	Studies that include interventions that use exercise only to reduce weight
Commercial weight loss program	Studies that compare the efficacy of commercial weight loss programs
Web-based/app-based/text messaging/ electronic device	Studies that test a web-based platform or app-based platform or text messaging or electronic device to deliver a weight loss intervention.
Self-Weighing	Studies that test the implication of self-weighing on weight loss
Counseling/additional therapeutic contact/ behavioral therapy/lifestyle intervention	Studies that use additional therapeutic contact or behavioral therapy or lifestyle interventions as the focus of a weight loss intervention
Community-based intervention	Studies that are based in local communities and a developed based on cultural or social interactions
Family-based intervention	Studies that are based on the participation of various family members within the one intervention
Dietary guidelines	Studies that test the efficacy of published clinical guidelines on weight loss
Information only	Studies that provide participants with written information only as an intervention to support weight loss
Meal frequency	Studies that investigate the frequency of meals and the impact on weight loss

**Table 2:** Results by themes and broad classifications.

Themes	No of Studies				Ranking	Broad classification	Broad classification
	Australia	Canada	UK	Total: Themes			
Macronutrients	6	2	1	9	1	Scientific advancement/ investigating biological interactions and weight loss	22
Caloric restriction +/- exercise	1	7	0	8	2		
Micronutrients	1	2	0	3	6		
Exercise only	0	1	1	2	8		
Commercial weight loss program	1^a^	5	2^a^	7	4	New technology or commercial opportunities	13
Web-based/ app-based/ text messaging/ electronic device	4^a^	0	3^a^	6	5		
Self-weighing	0	0	2	2	8	Psychological/ behavioral theories	10
Counseling/ additional therapeutic contact/ behavioral therapy/lifestyle intervention	4	0	4	8	2		
Community-based intervention	2	0	1	3	6	Community-based intervention	5
Family-based intervention	1	0	1	2	8		
Dietary guidelines	1	0	0	1	11	Efficacy of information or guidelines	3
Information only	0	0	1	1	11		
Meal frequency	0	1	0	1	11		

^a^Note: One study was conducted in both Australia and the United Kingdom.

**Table 3:** Indicators of Community Engagement (CE) within studies reviewed.

Themes	CE Development	CE Future	CE Acknowledgement
No. of Studies		
Macronutrients	0	0	2
Caloric restriction +/- exercise	0	0	2
Micronutrients	0	0	0
Exercise only	0	0	1
Commercial weight loss program	0	0	1
Web-based/ app-based/ text messaging/electronic device	0	1	3
Self-weighing	0	0	0
Counseling/additional therapeutic contact/ behavioral therapy/lifestyle intervention	0	1	4
Community-based intervention	1	1	2
Family-based intervention	0	1	1
Dietary guidelines	0	0	1
Information only	1	0	0
Meal frequency	0	0	0
Total	2	4	17

## Discussion

The majority of the studies reviewed were conducted in response to “Scientific advancements/Investigating biological interactions and weight loss” and “New technologies or commercial opportunities”, that is, they were primarily advancing and testing new knowledge (such as micronutrient or macronutrient involvement in weight loss) or tools (such as the Internet and electronic devices to deliver interventions). This is to be expected and encouraged in an evidence-based health sector. What is of interest is that there were only a few studies that were community-based and very few studies that reported significant community engagement. While the subject matter for this review was interventions that aim to reduce weight and the results cannot be generalized to all public health areas, it gives us an indication that in public health research, the notion of community engagement and empowerment has not been fully leveraged.

This is important because the foundation of public health revolves around empowerment, community involvement, a multidisciplinary alliance and achieving equity in health [17]. Empowerment refers to the ability of people to acquire an understanding and control over personal, social and economic influences on their health so that they are able to act in a way that will improve their life situation [18]. These are all factors that are difficult to measure and in the context of public health it is a challenge as it may not always be possible to report community engagement and empowerment in a way that satisfies traditional impact measures.

Another challenge is that the emphasis on empowerment is often in contrast with equally influential notions of evidence-based decision making including cost-effectiveness and population health approaches. This is largely driven by stakeholders and decision makers often being more concerned with the ability to measure outcome and define empirical success rather than the value that the target population places on the initiative itself [19]. In the context of this review, it should be noted therefore that there may have been more community engagement within the studies reviewed, but it was not reported as it is not generally valued or requested in scientific literature.

It appears then that a key challenge in public health is to build high quality and appropriate standards for evidence-based evaluation that the community, researchers and policy makers can mutually benefit from.

In the public health setting, the promotion of health is defined as a process in which individuals are able to increase control over their health resulting in an improvement in their life [20]. While it is not a new document, the Ottawa Charter continues to provide public health practitioners with guidance from a combination of its five action areas. Within the five action areas there are two key elements that are particularly relevant to public health policy. The first is to “Build healthy public policy”, and the second is to “Strengthen Community Actions”. The first element relates to the regulatory aspects of public health where policies and laws are created to enforce health promotion initiatives while the second element relates to empowerment and the ability of communities and patients to set priorities, make decisions, plan and implement programs that help them to improve their health outcomes. While these are both extremely important elements, they are potentially conflicting and it is not clear whether they can coexist in the context of modern public health, as was evidenced in this review where a number of high quality weight loss studies reported detailed clinical and policy-related outcomes. However, the vast majority of studies did not report or measure community engagement.

When we look at the results of the community engagement indicators in this review, there were only five studies that demonstrated an effort to engage patients in the development of their research protocol or future research initiatives. It is clear that this is an area that researchers have not completely embraced as part of their research process, yet patients and the general public are demanding an increased level of accountability from health professionals and policy makers regarding allocations of health resources by governments and health care providers [21-25]. This is important because research that includes collaboration between health professionals, knowledge through research, and drawing upon patients and community members’ knowledge about their health, safety and well-being are most effective, particularly when they are complemented with an analysis of the needs and expectations of the community [26-28]. Acknowledging the differences in community needs and expectations may firstly avoid the development of a problematic or inappropriate health policy or initiative [29], and furthermore can assist in creating a supportive environment and improve an individual’s ability to access all appropriate and available services and treatments [30-35].

There are, however, conflicting paradigms in health that challenge our ability to engage the community and drive research and policy that addresses individual needs. While population health approaches aim to deliver services and initiatives that serve the greater population, it is often at the risk of bypassing minority groups and potentially increasing the gap in health inequality [26,36]. Public health has progressed from a largely reactive model, to a preventative model. The next step is to make it a more proactive movement. That is not to say that it should not be reactive or preventative, but rather a combination of various elements of previous public health models. The differences between the old and the new public health are relatively subtle and are in many ways the result of the different context and environments in which public health exists [37]. Moving forward, the sector will need to acknowledge that there are many determinants of health and risk factors, some of which will be restricted to small subpopulations, which can be addressed by also using multi-sectoral and innovative partnerships to implement practical work plans [27,28].

This evolution means that public health professionals will be required to have expertise not only in health, but knowledge of a wider range of disciplines, an understanding of community engagement methods and incorporate a multidisciplinary approach to health in their decision making. Another explanation for the poor level of community engagement in this review may therefore be a lack of support and training for researchers to implement community engagement activities. This is important because those who create health policies are also now required to take into account the varying contexts that affect health outcomes [38] and if the community view is not included in the research that informs policies, the ability to make informed decisions may be compromised. This evolution has certainly created a more complex platform for public health; however, it also provides valuable opportunities for collaboration with an extended range of stakeholders including patients and the community, who can contribute additional knowledge, experience and set the expectations of public health initiatives.

### Conclusion

This review provides a demonstrative snapshot of the level of community engagement in one area of public health research. While it is not common to all areas of public health, it is clear there are many drivers of public health initiatives and that scientific and technological advancements are the primary drivers of research. However, these new research initiatives have largely been undertaken in the absence of community engagement. Development and evaluation of research and public health initiatives need to adapt to a wider range of stakeholders including looking for best practice community engagement, embracing new prospects for collaboration, developing new and equitable patient and community engagement platforms and taking into consideration the more complex social environment as well as individual needs.

## Abbreviations

CE: community engagement
